# An efficient data structure for calculation of unstructured T-spline surfaces

**DOI:** 10.1186/s42492-019-0010-0

**Published:** 2019-05-22

**Authors:** Wei Wang, Yang Zhang, Xiaoxiao Du, Gang Zhao

**Affiliations:** School of Mechanical Engineering and Automation, Beihang University, Beijing, 100191 People’s Republic of China

**Keywords:** T-splines, Non-uniform rational B-splines, Unstructured T-mesh, Extraordinary points

## Abstract

To overcome the topological constraints of non-uniform rational B-splines, T-splines have been proposed to define the freeform surfaces. The introduction of T-junctions and extraordinary points makes it possible to represent arbitrarily shaped models by a single T-spline surface. Whereas, the complexity and flexibility of topology structure bring difficulty in programming, which have caused a great obstacle for the development and application of T-spline technologies. So far, research literatures concerning T-spline data structures compatible with extraordinary points are very scarce. In this paper, an efficient data structure for calculation of unstructured T-spline surfaces is developed, by which any complex T-spline surface models can be easily and efficiently computed. Several unstructured T-spline surface models are calculated and visualized in our prototype system to verify the validity of the proposed method.

## Introduction

With a series of excellent mathematical and algorithmic properties, non-uniform rational B-splines (NURBS) has been widely used in the field of computer aided geometric design for representing curves and surfaces. Nevertheless, in modern industry, complex engineering models comprised of multiple NURBS patches are always not watertight because of the existence of gaps and overlaps along the interfaces of trimmed NURBS surfaces. Thus, T-splines were firstly proposed by Sederberg et al. [1, 2] in 2003 to conquer the limitations of NURBS in practical engineering applications.

As a generalization of NURBS, T-splines introduce T-junctions and extraordinary points into its control mesh. Theoretically, a T-spline surface can represent any arbitrarily shaped model no matter how complicated its topology structure is. Compared with NURBS, the advantages of T-splines can be reflected in the following aspects. Firstly, a NURBS surface is defined in a rectangular topological grid. It requires a large number of superfluous control points to maintain the topological shape while implementing refinement. This shortcoming can be overcome by T-splines which can achieve local refinement without introducing an entire row of control points. In addition, it is difficult to represent a complex model with a single NURBS surface and the gaps along the common boundary of two NURBS surfaces are unavoidable. T-splines provide a promising way to breakdown these barriers. In ref.  [3], multiple trimmed NURBS patches are merged into a single watertight T-spline surface. Li et al.[4] studied the linear independence of T-spline blending functions and proposed the notion of analysis-suitable T-splines. Analysis-suitable T-splines satisfy a simple topological requirement and their blending functions are linear independent [4–6]. So far, T-splines have been used in many fields such as geometric modeling [7–9], isogeometric analysis [10–15] and shape optimization [16–18].

In complex T-spline models, the extraordinary points are always indispensable. T-splines containing extraordinary points are called the unstructured T-splines [14]. When encountering an unstructured T-spline surface, the knot interval vectors about the vertexes around the extraordinary points are ambiguous. More details about the concept of extraordinary points are presented in section 2. Some methods have been developed to deal with the problems caused by extraordinary points [14, 19, 20]. In the template method proposed by Wang et al. [19], gap-free T-spline surfaces are generated by inserting zero-interval edges around the extraordinary points. Liu et al. [20] proposed a knot interval duplication and optimization method to obtain local knot vectors. In ref. [14], Scott et al. introduced a linear interpolation scheme to calculate Bézier control points from T-spline control points, which is easy to understand and implement.

Since T-spline surfaces have flexible topology, constructing a robust and efficient data structures of T-splines for storing and further data processing is a challenging topic. Asche et al. [21] presented a T-spline data structure implementation based on a half-edge (HE) data structure and implemented the algorithms with CGAL geometry programming library. Lin et al. [22] developed the so-called extended T-mesh which can be represented in an *obj*-like format file and converted into the face-edge-vertex data structure conveniently. With this method, each vertex in the extended T-mesh has a knot coordinates, which cannot solve the situtation with extraordinary points. Xiao et al. [23] also proposed a set of new T-spline data models to obtain better data storing and operating efficiencies. However, all the T-spline data structures mentioned above cannot deal with the T-splines with extraordinary points, i.e., unstructured T-splines. To the best knowledge of the authors, there are no public research papers or open sources which directly present the suitable approaches to handle the unstructured T-splines from a view of programming implementation.

In this paper, a new data structure for the unstructured T-splines is proposed. An efficient local parameterization algorithm which can accelerate the computation of T-spline surface is also presented. Finally, several testing examples of unstructured T-splines are demonstrated to show the validity of the proposed data structures and algorithm.

The rest of the paper is organized as follows. In section 2, we give a brief introduction of T-splines and explain the concept of extraordinary points. Section 3 is devoted to present the new data structure. In section 4, we give the local parameterization algorithm. Finally, complicated T-spline models are demonstrated in section 5.

## T-splines

A brief introduction of T-splines is reviewed in this section. We give a description of some symbols and notations appear below as well. In this paper, we only consider bicubic T-splines.

### T-mesh

The T-mesh that contains the underlying topology information is a fundamental concept of a T-spline surface.

**Fig. 1:**
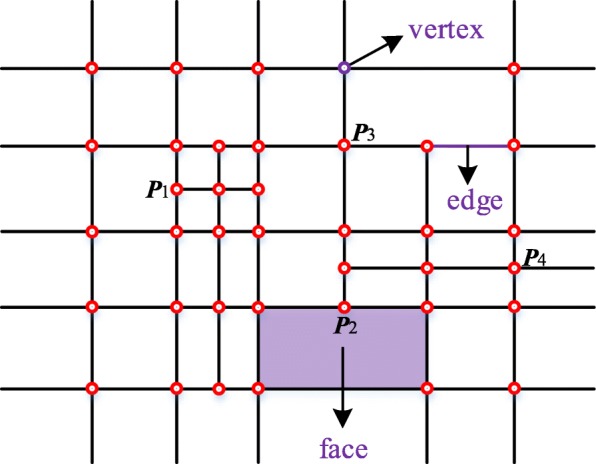
A T-mesh

As the example shown in Fig. 1, a T-mesh is composed of faces, edges and vertexes. For bicubic T-splines, a control point and related weight are aligned to each vertex in the T-mesh. The T-junctions such as ***P***_1_ and ***P***_2_ in Fig. 1 make the topological structure of T-splines very flexible. After a valid knot interval configuration being assigned to the edges, the topology information of T-splines is determined.

### T-spline blending function

Each vertex in a T-mesh corresponds to a T-spline blending function. We can construct the blending function from the knot interval sequences inferred from the T-mesh. These knot interval sequences are called local knot interval vectors.

**Fig. 2:**
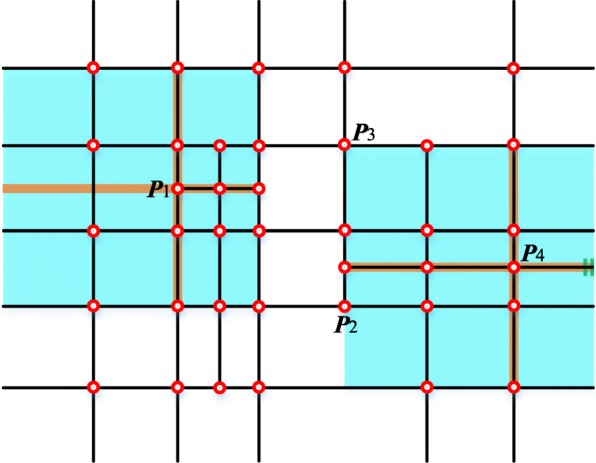
An example of the local knot interval vectors

The principles about how to deduce the local knot interval vectors from the T-mesh are presented in refs. [24, 25] in detail. In summary, as the brown lines shown in Fig. 2, marching through the T-mesh in four different topological directions until two vertices or perpendicular edges are detected, the knot interval vectors can be determined by the traversed distance. In normal conditions, the knot interval is set to be be 0 if a T-mesh boundary is crossed. The process of constructing local knot interval vectors for ***P***_1_ and ***P***_4_ is shown in Fig. 2.

As the example shown in Fig. 2, the influence domain which is called local blending function domains (the blue regions) corresponding to the control points can be defined after achieving the knot interval vectors. Then we can set up a local blending coordinate system attached to corresponding vertexes. If there exists no extraordinary point in a T-mesh, a larger global parametric coordinate system can be established which can help us compare different blending functions in a common coordinate system.

**Fig. 3:**
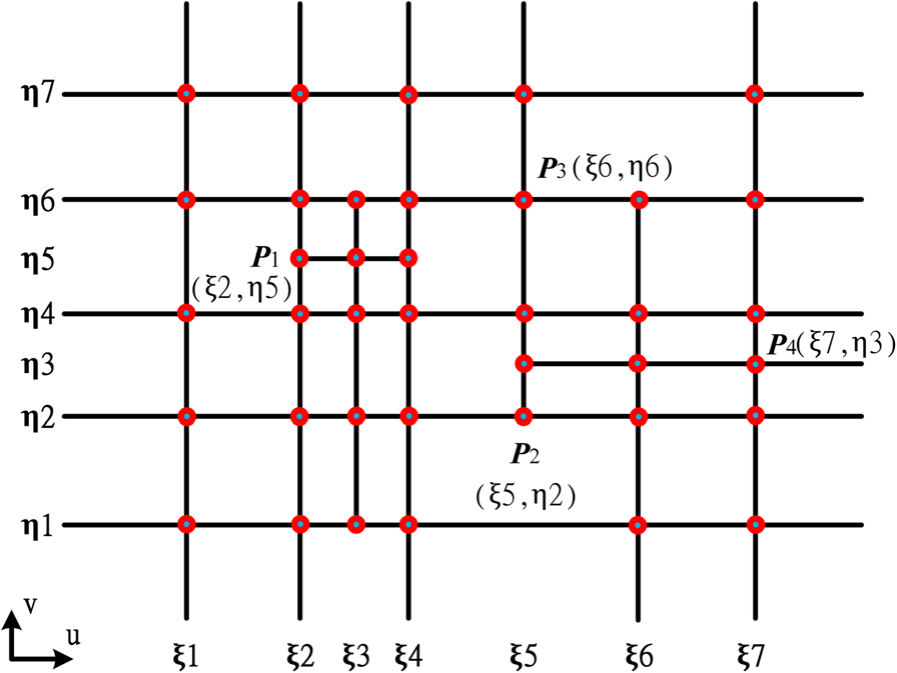
A global parametric coordinate system

The global parametric system of the T-mesh in Fig. 1 is shown in Fig. 3. Each vertex has only one pair of corresponding parameter coordinates. However, in the unstructured T-splines which contain extraordinary points, the situation is completely different and that is the main difficulty of constructing an efficient T-spline data structure.

The equation of a T-spline surface can be expressed as:

$$ \mathrm{T}=\frac{\sum_{i=1}^n{\mathbf{P}}_i{\upomega}_i{N}_i\left(\upmu, \upnu \right)}{\sum_{i=1}^n{\upomega}_i{N}_i\left(\upmu, \upnu \right)} $$

where **P**_*i*_ are control points, ω_*i*_ are weights, and *N*_*i*_ are blending functions, μ and ν are knot values.

### Extraordinary points

**Fig. 4:**
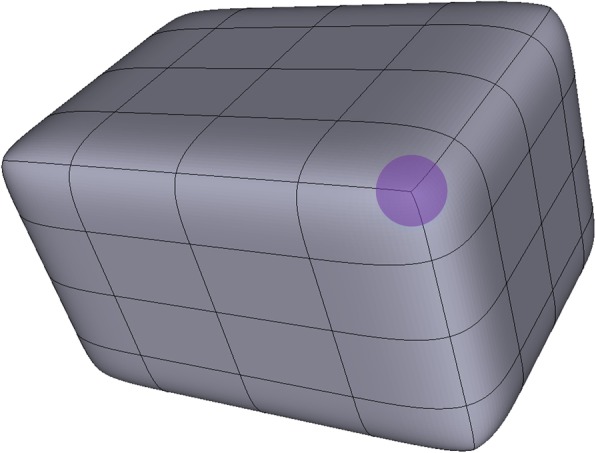
A cube T-spline model. An extraordinary point is marked in the purple region

In some T-spline models such as the cube shown in Fig. 4, the existence of extraordinary points is inevitable. There are eight extraordinary points on the corners of the cube.

**Fig. 5:**
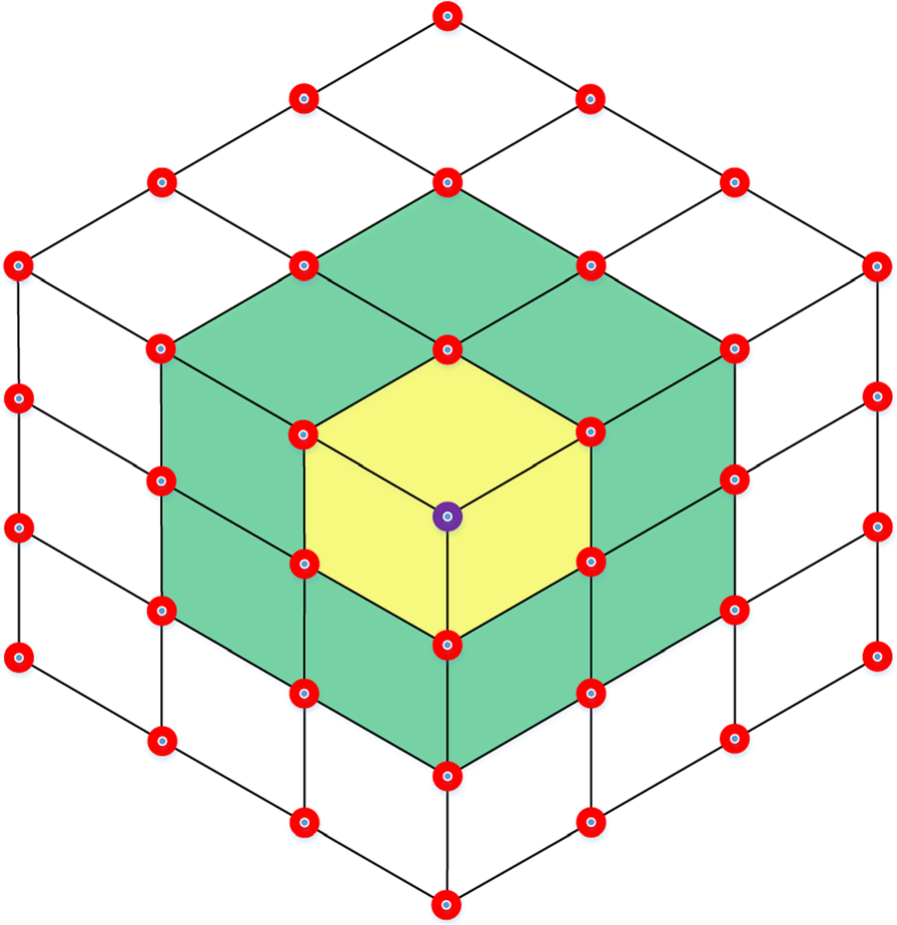
The T-mesh around the extraordinary point denoted by purple in Fig. 4

In a T-mesh, the valence of a vertex is the number of edges that touch the vertex. As the T-mesh shown in Fig. 2, the T-junctions ***P***_1_ and ***P***_2_ have three valences. The definition of extraordinary point is that an interior vertex that is not a T-junction and of which the valence is not equal to 4 [14]. Spoke edge is the edge connected to the extraordinary point. The one-ring neighborhood of a vertex refers to the T-mesh faces which touch the vertex. The faces that touch the one-ring neighborhood form the corresponding vertex’s two-ring neighborhood. The T-mesh around the extraordinary point in the purple region in Fig. 4 is shown in Fig. 5.

In Fig. 5, the valence of the extraordinary point marked by purple circle is 3. The one-ring neighborhood is represented by yellow and the two-ring neighborhood is represented by green. From the unstructured T-mesh, we can see that it is impossible to set up a common global coordinate system due to the existence of the extraordinary point, which brings problems to the knot interval vectors definition in their neighborhood.

## Data structure for unstructured T-splines

Owing to the fact that extraordinary points are unavoidable in complicated models, we should reconsider the existing T-spline data structures because with extraordinary point appearing it is impossible to assign each vertex a parameter coordinate in a common global parametric coordinate system.

The new data structure we proposed is inspired by the classical HE data structures. Since this data structure provides efficient retrieval of the topological information associated with the mesh, we can make some modifications to it to meet the requirements of storage and computation of the unstructured T-splines.

**Fig. 6:**
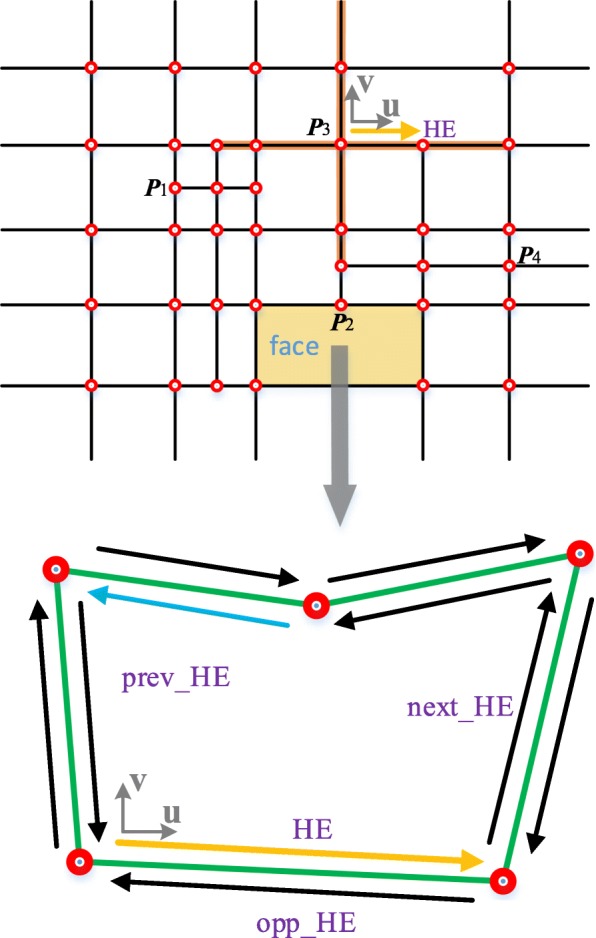
Schematic of the proposed data structure

The schematic of the proposed data structure (Table 1) is illustrated in Fig. 6. The face marked in yellow region in the upper T-mesh is composed of five HE. The HEs are denoted by colorful arrows and the green lines represent edges. An edge corresponds to two opposite HEs. Each HE starts from a vertex. What calls for special attention is that in the unstructured T-mesh, a HE doesn’t have a specific direction and a vertex doesn’t have a corresponding global parameter coordinate. This is the main difference between the proposed data structure and those constructed in a global coordinate system.

**Table 1:**
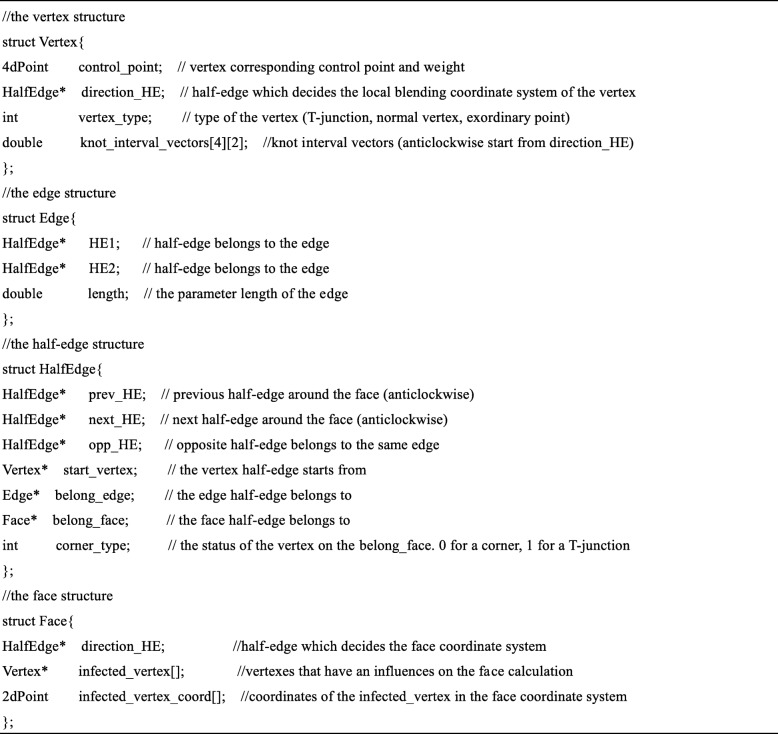
The data structure for the unstructured T-splines

To facilitate the calculation of the faces in an unstructured T-mesh, a local coordinate system is established for each face. As the example shown in Fig. 6, the HE denoted by yellow is chosen to act as the *u* direction of the face coordinate system. To make the calculation of the faces more convenient, we can choose any HEs denoted by a black arrow inside the face to set up the face parametric coordinate system, with the exception of the one starts from a T-junction (the HE denoted by blue in Fig. 6).

In order to save in-time computations of the T-spline surface, some redundant data or pointers are stored in the data structure. In order to search the vertexes that have an impact on the faces in the T-mesh, it is essential to select a HE to set up a local blending coordinate system for each vertex (such as the local blending coordinate system of ***P***_3_ in Fig. 6). For the faces, the infected vertexes and their relative coordinates in face coordinate systems are stored in the data structure.

## Computation of the unstructured T-splines

An efficient data structure must not only be flexible for data storing but also suitable for the development of related algorithms. In this section, we present an efficient algorithm for the computation of the unstructured T-splines based on the proposed data structure.

Because the faces in a T-mesh have one-to-one mapping relations with the patches in a T-spline surface, we can tessellation the T-spline surface face by face. The computation of the T-spline surfaces can be summarized as the following steps.

Step 1: Load the T-spline models. In this paper, the T-spline models are constructed in Rhinoceros and saved as *TSM*-files.

Step 2: Construct the T-spline data structures.

Step 3: For each vertex, establish a local blending coordinate system in the parametric domain after obtaining the local knot interval vectors, as the example shown in Fig. 2.

Step 4: Find all the faces that overlap the local parametric domain of the vertex. Then obtain the coordinate of the vertex in different face parametric coordinate systems.

Step 5: For each face find in step 4, add the vertex and coordinate into the face data structure.

Step 6: Compute the T-spline surface face by face by the computation formula.

In the unstructured T-splines, most of the time is spent on step 4 during the surface computation due to the lack of a global parametric coordinate system. Here we give an efficient algorithm called local parameterization (Table 2) which can improve the efficiency of the calculation of T-spline surfaces.

**Table 2:**
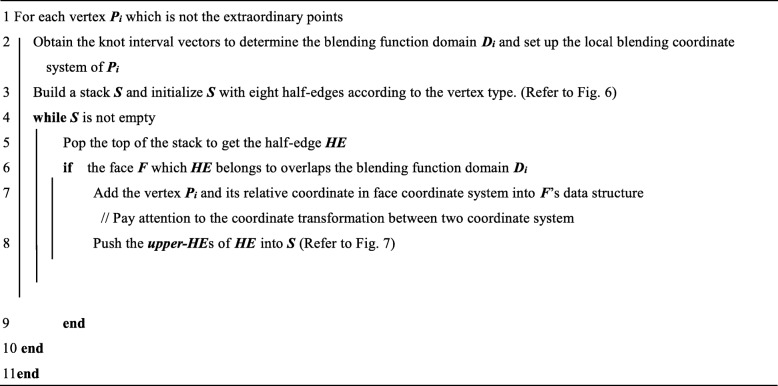
Local parameterization algorithm

**Fig. 7:**
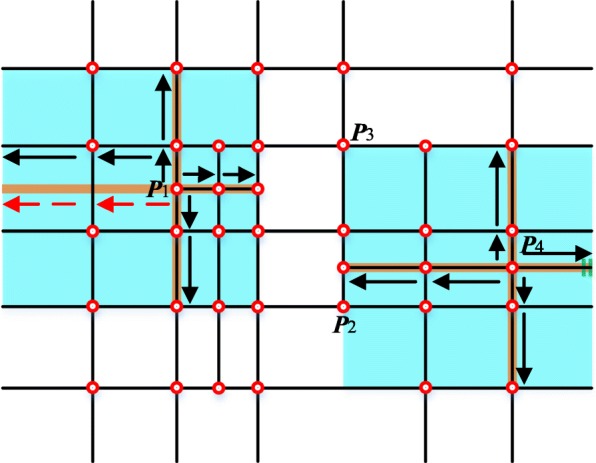
Initial half-edges in the local parametric algorithm

The main idea of the local parameterization algorithm is to traverse all faces in the local blending function domain of each vertex and then obtain the coordinate of the specific vertex in face coordinate systems. In the algorithm, the whole traversal process of the domain is realized from four directions represented by eight HEs. These HEs can be obtained during the procedure of obtaining the local knot interval vectors. If the vertex is a T-junction such as ***P***_1_ shown in Fig. 7, we should change the virtual HE marked by red dashed arrows to the black solid arrows. The eight arrows (in black) are the initial HEs.

To complete the traverse process, if a half-edge ***HE*** is popped from the stack ***S*** in line 3 of the algorithm, we should judge whether the face ***F*** it belongs to overlap the blending function domain ***D***_***i***_ of the vertex ***P***_***i***_. In that case, the vertex ***P***_***i***_ and its coordinate in face ***F***’s coordinate system should be added into the data structure of ***F*** firstly. Then the half-edges called ***upper-HE***s which parallel to ***HE*** and belong to the faces above ***F*** should be pushed into the stack ***S***. In this way, we can make sure that all faces in the blending function domain can be traversed without any omissions.

**Fig. 8:**
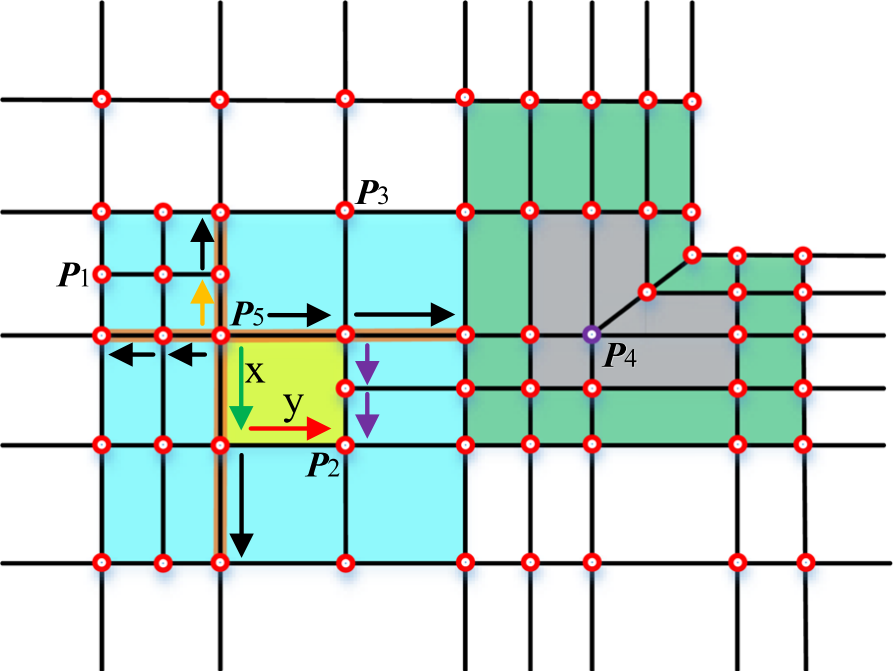
Schematic of the local parametric algorithm

As the unstructured T-spline with an extraordinary point ***P***_4_ shown in Fig. 8, assume that the orange arrow starting from ***P***_5_ is the *u* direction of the local blending coordinate system of ***P***_5_ and the red arrow represents the *u* direction of the face coordinate system denoted by yellow region. The parameter length of the two HEs marked by green and red are *x* and *y*, respectively. After the initialization, if the HE denoted by green is popped up from the stack, we can see the face (yellow region) it belongs to obviously overlap the local blending domain marked in blue. In the face coordinate system, the parametric coordinate of ***P***_*5*_ is (0, x). Then ***P***_5_ and the coordinate should be added into the face data structure because ***P***_5_ has an effect on the calculation of the yellow face. According to the content described in line 8 of the local parameterization algorithm, two HEs denoted by purple arrows (***upper-HE***s mentioned above) should be pushed into the stack. Thus, we can accomplish the algorithm by repeating this process. The parameter coordinate of the vertex ***P***_5_ in different face coordinate systems is easy to obtain through the connections between the half-edges.

In the neighborhood of the extraordinary points (the grey and green regions around ***P***_5_ in Fig. 8), the knot interval vectors definition is a little tricky. Many literatures have studied on this topic [19, 20, 26]. In order to ensure continuity of the elements near the extraordinary points for further application such as isogeometric analysis, in this paper, we choose the method proposed by Scott et al. [14] to solve this problem. Through the method described in ref. [14], two-ring neighborhood elements are *C*^2^ with adjoining three-ring neighborhood elements, and *C*^1^ with their other neighbors; and one-ring neighborhood elements are *G*^1^ with adjoining one-ring neighborhood elements and *C*^1^ with adjoining two-ring neighborhood elements.

**Fig. 9:**
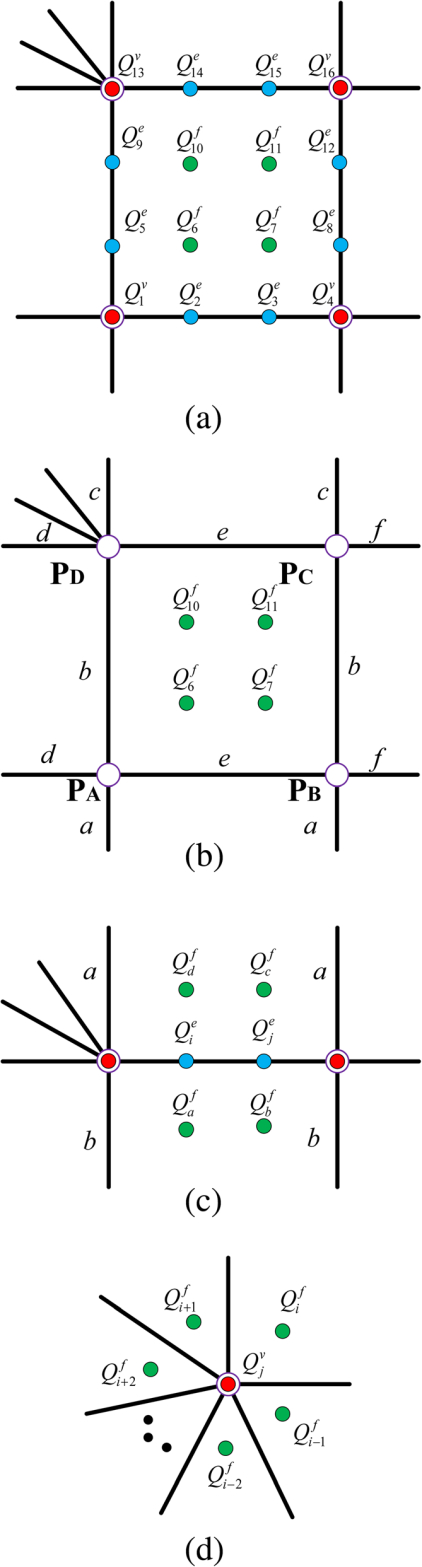
Scheme of calculating two-ring neighborhood of the extraordinary points [11]

In the two-ring neighborhood of an extraordinary point, the faces can be represented by the linear combinations of the T-spline control points. They can be calculated as patches of Bézier elements and the procedure is simply described in Fig. 9. Each bicubic elements contains 16 Bézier control points which can be classified into face points (solid green circles), edge points (solid blue circles) and vertex points (solid red circles). Each face point denoted by a superscript *f* can be represented in terms of T-spline control points (denoted by purple circles). Each edge point denoted by a superscript *e* is written in terms of face points and each vertex point denoted by a superscript *v* is represent by the face points. The formulas are defined as.

$$ {\displaystyle \begin{array}{l}{Q}_6^f=\left(\frac{b+c}{a+b+c}\right)\left(\frac{e+f}{d+e+f}\right){\boldsymbol{P}}_A+\left(\frac{b+c}{a+b+c}\right)\left(\frac{d}{d+e+f}\right){\boldsymbol{P}}_B\\ {}+\left(\frac{a}{a+b+c}\right)\left(\frac{d}{d+e+f}\right){\boldsymbol{P}}_C+\left(\frac{a}{a+b+c}\right)\left(\frac{e+f}{d+e+f}\right){\boldsymbol{P}}_D\\ {}{Q}_7^f=\left(\frac{b+c}{a+b+c}\right)\left(\frac{f}{d+e+f}\right){\boldsymbol{P}}_A+\left(\frac{b+c}{a+b+c}\right)\left(\frac{d+e}{d+e+f}\right){\boldsymbol{P}}_B\\ {}+\left(\frac{a}{a+b+c}\right)\left(\frac{d+e}{d+e+f}\right){\boldsymbol{P}}_C+\left(\frac{a}{a+b+c}\right)\left(\frac{e}{d+e+f}\right){\boldsymbol{P}}_D\\ {}{Q}_{10}^f=\left(\frac{c}{a+b+c}\right)\left(\frac{e+f}{d+e+f}\right){\boldsymbol{P}}_A+\left(\frac{c}{a+b+c}\right)\left(\frac{d}{d+e+f}\right){\boldsymbol{P}}_B\\ {}+\left(\frac{a+b}{a+b+c}\right)\left(\frac{d}{d+e+f}\right){\boldsymbol{P}}_C+\left(\frac{a+b}{a+b+c}\right)\left(\frac{e+f}{d+e+f}\right){\boldsymbol{P}}_D\\ {}\end{array}} $$

$$ {\displaystyle \begin{array}{l}{Q}_{11}^f=\left(\frac{c}{a+b+c}\right)\left(\frac{f}{d+e+f}\right){\boldsymbol{P}}_A+\left(\frac{c}{a+b+c}\right)\left(\frac{d+e}{d+e+f}\right){\boldsymbol{P}}_B\\ {}+\left(\frac{a+b}{a+b+c}\right)\left(\frac{d+e}{d+e+f}\right){\boldsymbol{P}}_C+\left(\frac{a+b}{a+b+c}\right)\left(\frac{f}{d+e+f}\right){\boldsymbol{P}}_D\\ {}{Q}_i^e=\left(\frac{a}{a+b}\right){\mathrm{Q}}_a^f+\left(\frac{b}{a+b}\right){\mathrm{Q}}_d^f\\ {}{Q}_j^e=\left(\frac{a}{a+b}\right){\mathrm{Q}}_b^f+\left(\frac{b}{a+b}\right){\mathrm{Q}}_c^f\\ {}{Q}_j^v=\sum \limits_{i=1}^N\left(\frac{a^{i-1}}{a^{i-1}+{a}^{i+1}}\right)\left(\frac{a^{i+2}}{a^i+{a}^{i+2}}\right){\mathrm{Q}}_i^f\end{array}} $$

## Results and discussion

**Fig. 10:**
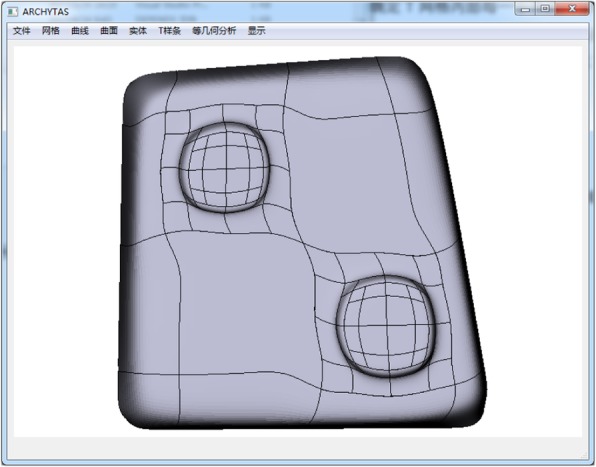
Gearbox cover

**Fig. 11:**
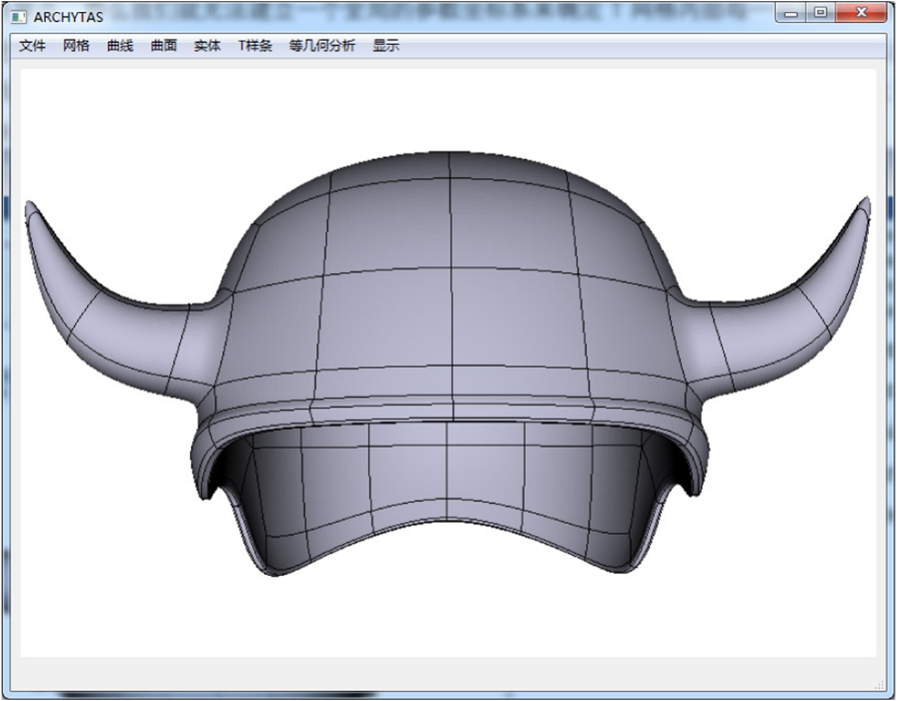
Helmet

**Fig. 12:**
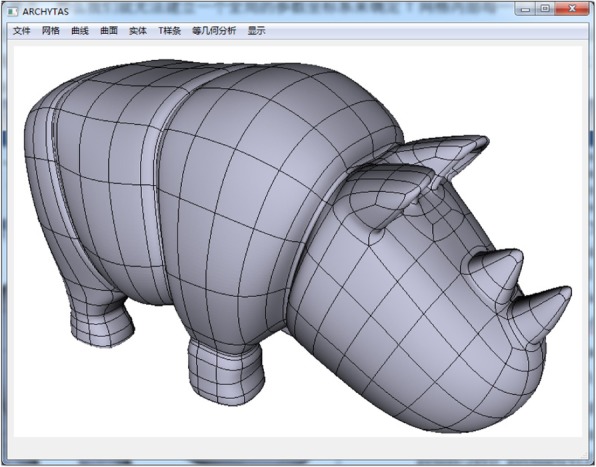
Rhino

**Fig. 13:**
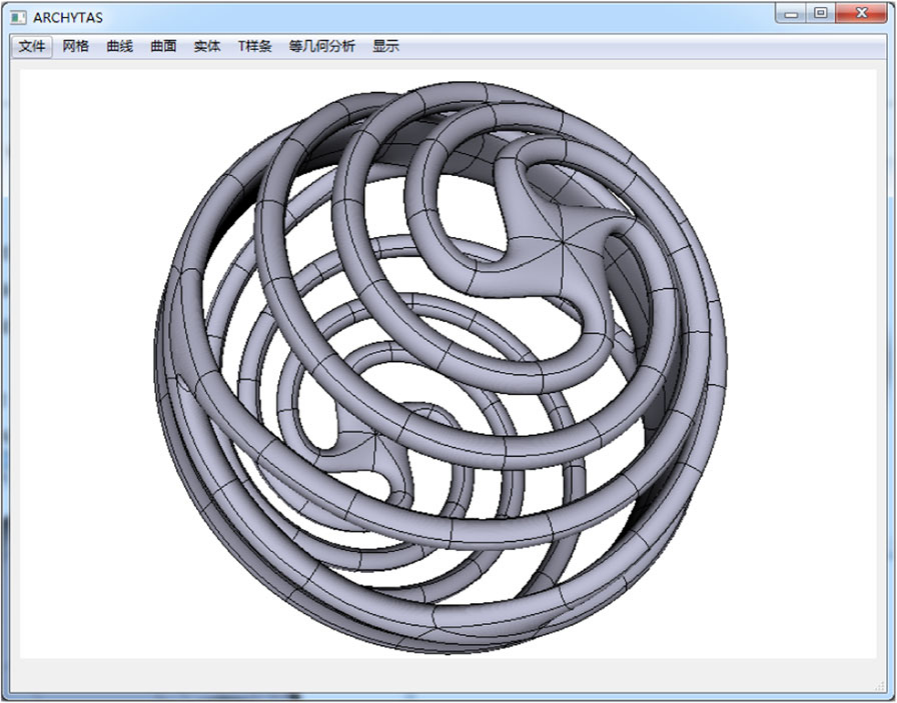
Spiral

In this section, as given in Figs. 10, 11, 12, and 13 some T-spline models are shown to verify the feasibility of the proposed data structures and the local parameterization algorithm. All the models are built from unstructured T-splines which include the extraordinary points except for the gearbox. We can download them from the official site of Rhinoceros [27].

## Conclusion

In this paper, an efficient data structure for the unstructured T-splines is proposed. With this data structure, the topology information of the T-splines can be accurately stored. In addition, a valid local parameterization algorithm which can improve the efficiency of the calculation of T-spline surfaces is developed. Some unstructured T-spline surface models are presented to verify the feasibility of data structures. All the data structures and algorithms presented in this paper have been implemented in our CAD/CAE/OPT integration software Archytas. In the future, the local refinement and the other correlative algorithms will be developed based on the proposed data structures.

## Abbreviations

HE: Half-edges
NURBS: Non-uniform rational B-splines
